# *In Vivo* Tracking of Human Neural Stem Cells with ^19^F Magnetic Resonance Imaging

**DOI:** 10.1371/journal.pone.0029040

**Published:** 2011-12-28

**Authors:** Philipp Boehm-Sturm, Luam Mengler, Stefan Wecker, Mathias Hoehn, Therése Kallur

**Affiliations:** 1 In-Vivo-NMR Laboratory, Max Planck Institute for Neurological Research, Cologne, Germany; 2 Medres – Medical Research GmbH, Cologne, Germany; Julius-Maximilians-Universität Würzburg, Germany

## Abstract

**Background:**

Magnetic resonance imaging (MRI) is a promising tool for monitoring stem cell-based therapy. Conventionally, cells loaded with ironoxide nanoparticles appear hypointense on MR images. However, the contrast generated by ironoxide labeled cells is neither specific due to ambiguous background nor quantitative. A strategy to overcome these drawbacks is ^19^F MRI of cells labeled with perfluorocarbons. We show here for the first time that human neural stem cells (NSCs), a promising candidate for clinical translation of stem cell-based therapy of the brain, can be labeled with ^19^F as well as detected and quantified *in vitro* and after brain implantation.

**Methodology/Principal Findings:**

Human NSCs were labeled with perfluoropolyether (PFPE). Labeling efficacy was assessed with ^19^F MR spectroscopy, influence of the label on cell phenotypes studied by immunocytochemistry. For *in vitro* MRI, NSCs were suspended in gelatin at varying densities. For *in vivo* experiments, labeled NSCs were implanted into the striatum of mice. A decrease of cell viability was observed directly after incubation with PFPE, which re-normalized after 7 days in culture of the replated cells. No label-related changes in the numbers of Ki67, nestin, GFAP, or βIII-tubulin+ cells were detected, both *in vitro* and on histological sections. We found that 1,000 NSCs were needed to accumulate in one image voxel to generate significant signal-to-noise ratio *in vitro*. A detection limit of ∼10,000 cells was found *in vivo*. The location and density of human cells (hunu+) on histological sections correlated well with observations in the ^19^F MR images.

**Conclusion/Significance:**

Our results show that NSCs can be efficiently labeled with ^19^F with little effects on viability or proliferation and differentiation capacity. We show for the first time that ^19^F MRI can be utilized for tracking human NSCs in brain implantation studies, which ultimately aim for restoring loss of function after acute and neurodegenerative disorders.

## Introduction

To achieve translation of experimental stem cell-based therapy into the clinic, non-invasive imaging modalities are necessary tools. One such modality, magnetic resonance imaging (MRI), provides true three-dimensional data at high spatial resolution, enabling good detection of even small cell numbers in the living, intact individual. Commonly, contrast is achieved through *in vitro* labeling of cells with superparamagnetic iron oxide (SPIO) nanoparticles [1], [2], [3]. Although even single cells can be detected [4] with this procedure, the contrast generated by iron oxide labeled cells can easily be confounded with other sources such as bleedings or blood vessels [5]. Furthermore, since contrast is achieved indirectly through disturbances of the local magnetic field experienced by surrounding hydrogen nuclei, quantification of the number of cells *in vivo* is questionable [6].

A rapidly emerging field to overcome these drawbacks of ambiguity of contrast assignment and cell quantification is cell labeling with perfluorocarbon (PFC) nano-emulsions, which can be detected with ^19^F MRI [7], [8], [9]. The ^19^F nucleus is particularly suitable for labeling as its relative MR sensitivity is only 17% less than that of ^1^H. Furthermore, the signal intensity is directly proportional to the number of accumulated ^19^F, hence, allowing *in vivo* quantification of ^19^F labeled cells [10]. In addition, since the level of background ^19^F signal in host tissue is virtually absent [10], overlaying the ^19^F image on an anatomical ^1^H image allows for unambiguous, quantitative tracking of labeled cells *in vivo*. However, compared to labeling and tracking with metal-based contrast agents the technique is considerably less sensitive requiring a large amount of ^19^F to accumulate in order to generate sufficient signal-to-noise ratio (SNR).

The strategy of ^19^F cell labeling has already been applied to monitor cells during pathological conditions, e.g. umbilical cord blood cell localization in tumor-bearing mice [11], T-cell migration in murine models of diabetes [12], and local inflammation [13]. More recently, PFCs have proven useful in ^19^F MRI studies of inflammatory response to cerebral and cardiac ischemia [14] and *in vivo* measurements of intracellular pO_2_ of glioblastoma cells in response to chemotherapy [15].

In experimental models of human neurodegenerative diseases cell therapies have shown that neuronal replacement and partial repair of damaged brain circuitry is possible [16]. For a successful clinical translation, only cells of human origin will be needed, and one source of human cells are neural stem cells (NSCs) [17]. NSCs derived from the human fetal striatum have been expanded with both maintained normal karyotype and high capacity to generate different neuronal phenotypes for a long time *in vitro* [18], [19]. Upon implantation, these cells survived in the stroke-damaged rat striatum, migrated towards the injury, and differentiated into mature neurons without tumor formation [18].

Since these cells represent a relevant cell source for clinical translation, the purpose of the present study was to establish a platform to visualize NSCs *in vivo* after intracerebral implantation with ^19^F MRI. Monitoring the spatio-temporal dynamics of NSCs grafted into the brain requires the ability to detect even low cell numbers with high spatial resolution, since pathology-related migration processes of interest may take place on a small scale, the graft size may be diluted due to these processes or be initially relatively small. A first report has already indicated that it is feasible to detect fluorine labeled, immortalized, murine neural progenitor cells in the healthy mouse brain with ^19^F MRI [20]. The current study is the first to show that these results can be extended to the tracking of human NSCs. Novelties of our experiments compared to the previous studies on ^19^F MRI of stem cells [11], [20] include i) the use of a clinically relevant source of neural stem cells and detection with *in vivo* ^19^F MRI in a proof-of-concept, ii) a conservative estimate of cell detection limits for preclinical ^19^F MRI studies of neural stem cell implantation, and iii) a careful assessment of potential adverse effects of the ^19^F marker on cell viability and function both *in vitro* and *in vivo* over a period of at least one week. We show that under optimized preclinical conditions low numbers of cells can be detected *in vivo* at good resolution and within acceptable scan time. Our results suggest that ^19^F MRI may prove useful in monitoring implanted cells in models of neurodegeneration, thus allowing the optimization of preclinical protocols of graft induced brain repair.

## Materials and Methods

### Culturing of human neural stem cells

NSCs were obtained from the ganglionic eminences of an 8-week old aborted human fetus from Malmö/Lund University Hospitals, according to the guidelines approved by the Lund/Malmö Ethical Committee. The full characterization of these NSCs, derived from the human fetal striatum, has previously been described in detail [19]. Briefly, after microdissection and dissociation of the striatal tissue, cells were maintained at 37°C in a humidified atmosphere with 5% CO_2_. The neurosphere expansion medium (DMEM/F-12, Gibco, Grand Island, USA; l-glutamine, 2.92 g/100 mL; HEPES, 23.8 mg/100 mL; NaHCO_3_, 7.5%; glucose, 0.6%; and heparin, 2%, all from Sigma–Aldrich, Hamburg, Germany) contained N-2 supplement (1%, Gibco), human leukemia inhibitory factor (10 ng/mL, Sigma–Aldrich), epidermal growth factor, 20 ng/mL and fibroblast growth factor, 10 ng/mL (both from Peprotech, Hamburg, Germany).

### Labeling cells with ^19^F marker and determination of intracellular ^19^F content

A few hours prior to labeling, the small neurospheres were transferred to a Poly(2-hydroxyethyl methacrylate) (Poly-HEMA, Sigma-Aldrich) coated culture flask, in order to avoid strong attachment of the cells as a consequence of the label. We incubated cells with 5 µL, 15 µL, 50 µL, 100 µL, and 150 µL of a perfluoropolyether (PFPE) nano-emulsion (CELSENSE 1000, Celsense, Pittsburg, USA, 120 mg PFPE/ml, experimentally determined concentration of magnetically equivalent ^19^F c = 3.11 M)/mL culture medium to determine the optimal concentration in terms of fluorine uptake. In the end the emulsion was added at the empirically determined optimal (see Results) concentration of 100 µl/mL medium 3 days after the last passage directly to the NSC culture medium and cells were incubated for 36 hours. Subsequently, the cell suspension was spun down at 184× g for 5 min, the pellet was washed twice and finally resuspended in either fresh medium or potassium phosphate-buffered saline (KPBS) or Hank's Balanced Salt Solution (HBSS), depending on the application.

In order to determine the amount of intracellular ^19^F, 10 µl of a solution containing 10 mg KF/mL H_2_O (i.e. 1.04*10^18^ ^19^F spins) was added as an internal fluorine reference to the sample, which was then scanned with magnetic resonance spectroscopy (MRS, see below). The amount of fluorine was determined by comparing the integrated area of the PFPE main peak and the KF reference peak in the ^19^F MR spectra.

### Determination of in vitro cell detectability

To determine the limits of *in vitro* detectability with MRI, the ^19^F labeled, small neurospheres were spun down, washed in KPBS, fixed with 4% paraformaldehyde (PFA), washed again and resuspended in KPBS. The fixed, labeled neurospheres were either transferred to small tubes or injected at different cell concentrations into phantoms made of 2% gelatin in small, custom-made cups 22 mm in diameter for subsequent scans with MRI.

### Assessment of label influence on cell viability, proliferation and differentiation

Cell viability was determined in culture by trypan blue exclusion method before, shortly after, and at 7 days after labeling, and compared to unlabeled control cultures. Experiments were performed in triplicates. For determination of the cell phenotypes within the proliferating culture, ^19^F labeled neurospheres were plated on PLL coated 8-well chamber slides, allowed to attach for 3 h, then fixed and processed for immunocytochemistry. For determination of cell differentiation capacity, growth factors were removed and 1% fetal bovine serum (Gibco) was added. Labeled cells were differentiated for 9 days, then fixed and subsequently stained. Medium was changed every third day during experiments.

### Animal experiments

All experiments were conducted according to the guidelines laid out in the German Animal Welfare Act and approved by the local authorities (Landesamt für Natur, Umwelt und Verbraucherschutz Nordrhein-Westfalen) under permission number 9.93.2.10.31.07.048 (dated 22 May 2007). Four adult male CD 1 mice (bodyweight 37–42 g, Janvier, Le Genest Saint Isle, France) were used for the cell implantation and ^19^F MRI investigations. All experiments were performed under anesthesia. Animals were housed in cages under a 12 h light/12 h darkness cycle with access to food and water *ad libitum*.

### Implantation procedures

Human NSC cultures (passage 14) were labeled with ^19^F marker as stated above. At the day of implantation, ^19^F labeled neurospheres (diameter≈100 µm) were centrifuged and resuspended in HBSS (Gibco). The neurosphere suspension had a concentration of approximately 100,000 viable cells/µL and was kept on ice during the whole procedure. Mice were anesthetized with 1–2% isoflurane in a 70/30 nitrous oxide/oxygen mixture and placed in a stereotaxic frame. A feedback controlled system maintained the body temperature at 37°C (medres, Cologne, Germany). The non-labeled and ^19^F labeled neurosphere suspensions were injected using a Hamilton syringe into two deposits per hemisphere into the striatum (1.5 µL i.e. 150,000 cells per deposit) in two animals. Two further animals were injected bilaterally with two deposits of ^19^F labeled cells on the left and one deposit on the right hemisphere. The following coordinates were used: AP, +1.0, ML, ±2.0 mm from bregma, and DV, −3.0 and −2.0 mm from the brain surface. After each deposit, the needle was kept in place for 5 min before slow withdrawal. The wound was closed with suture and animals were allowed to recover in their cages. To suppress an immune reaction, animals received a subcutaneous injection of 20 mg/kg Cyclosporine A (Sigma-Aldrich) every second day starting 2 days before implantation.

### MRI acquisition

MRI and MRS were carried out on a Biospec 11.7 T/16 cm dedicated animal scanner system (Bruker BioSpin, Ettlingen, Germany) equipped with actively shielded gradient coils (BGA9S, 750 mT m^−1^, Bruker BioSpin). For radiofrequency transmission and reception, we used custom-built, inductively coupled, single-loop surface coils of 9 mm diameter for *in vitro* MRS, 25 mm diameter for *in vitro* MRI, and 20 mm diameter for *in vivo* MRI, all tunable from 470 MHz for the ^19^F resonance frequency up to 500 MHz for ^1^H imaging.

*In vitro* ^19^F MRS: For the *in vitro* quantification of ^19^F content with MRS, we used a spin-density weighted pulse-acquire spectroscopic sequence with short acquisition delay of 0.05 µs, and with long repetition time (TR) of 20 s to assure full relaxation recovery (90° rectangular hard pulse, duration/bandwidth (BW) = 0.01 ms/128 kHz, 163.8 ms acquisition window, spectral points/BW = 8192/50 kHz). We chose the number of averages (NA) dependent on the strength of PFPE signal, with typically NA = 30, leading to an acquisition time (TA) = 10 min. All measured ^19^F concentrations are presented with respect to the main PFPE frequency (−93.2 ppm), i.e. the chemical shift signal originating from the end group of the PFPE molecule was not taken into account.

Determination of relaxation times: To optimize sequence parameters, the relaxation times were measured with the 9 mm coil on a tube containing either the pure PFPE marker or the labeled cells. The temperature was kept at 37° throughout the experiment with a feedback controlled water system (medres). T_1_ was measured with a pulse-acquire saturation-recovery sequence (20 experiments, TR = 90.3 ms up to 20 s, pulse identical to *in vitro*, 66.7 ms acquisition window, spectral points/BW = 1000/15 kHz), T_2_ with a multi spin echo sequence (TR = 4 s, echo spacing 20 ms, field of view (FOV) = 16×16×2.5 mm^3^, 32×32 matrix, 15 echoes, NA = 4 (PFPE)/100 (cells), TA = 4 min/3:33 h). The spectral peak area or the signal in the multi-echo images 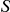 were then fitted with the software Origin (OriginLab Corporation, Northhampton, USA) to the function 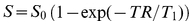 for T_1_ or 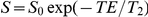 for T_2_, with the equilibrium signal 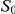.

*In vivo* ^19^F MRS: MRS was performed to determine the exact PFPE frequency *in vivo* using a pulse-acquire sequence identical to the *in vitro* experiments but with shorter TR (30° pulse, TR = 200 ms, NA = 3,000, TA = 10 min).

*In vitro* and *in vivo* ^1^H/^19^F imaging: Anatomical ^1^H imaging was performed with a turbo spin echo sequence (TR/effective echo time (TE_eff_) = 2200 ms/42.8 ms, 8 echoes per excitation, NA = 2, 10 consecutive, 1 mm thick slices, FOV = 1.92×1.92 cm^2^, 128×128 matrix, i.e. a resolution of 150×150×1000 µm^3^, TA = 1 min, BW = 50 kHz, linear phase encoding scheme) [21]. ^19^F images were acquired with the same sequence and matching geometry, but at lower in-plane resolution and lower BW (NA = 256, 48×48 matrix, i.e. a resolution of 400×400×1000 µm^3^, TA = 57 min, BW = 10 kHz). The transmission power needed for a 90° pulse was determined on the ^1^H channel on a 1 mm thick slice parallel to the coil through the ^19^F labeled cells and the same power was used after switching to the ^19^F frequency. Approximately the same power is needed for the ^1^H and the ^19^F 90° pulse, which we tested on a combined ^1^H/^19^F standard (diluted Trifluoroaceticacid, data not shown).

*In vivo* measurement protocol: *In vivo* ^19^F and ^1^H imaging was carried out 2 d after implantation for those mice that received non-labeled control cells, and 2 d and 6 d after implantation for the animals that were injected with labeled cells in both hemispheres. Mice were anesthetized with an intraperitoneal injection of a ketamine (120 mg/kg) and xylazine (8 mg/kg) mixture to avoid background signal from fluorinated inhalation of anesthesia gases. Anesthesia time was prolonged by subcutaneous administration of 30–60 mg/kg ketamine at 1 h after induction. Respiration rate was monitored using a pressure sensitive pad placed under the thorax, together with DASYlab (Measurement Computing, Norton, USA) software and body temperature was maintained at 37°C with an in-house feedback controlled system. Animals were fixed with ear bars in standard animal holders (Bruker BioSpin) and scanned with ^1^H MRI, ^19^F MRS, and ^19^F MRI. The total time of the imaging session did not exceed 1.5 h.

### Immunocytochemistry/immunohistochemistry

After the last MRI session, animals were deeply anesthetized and perfused transcardially with saline followed by 4% PFA. Brains were post-fixed overnight and then kept in 20% sucrose solution until they sunk. Thirty micrometer thick sections were cut in the coronal plane using a freezing microtome (Leica Microsystems, Wetzlar, Germany), and kept at −20°C in cryo-protective solution. Cells were fixed in 4% PFA for 15 min at room temperature followed by three rinses in KPBS. Before immunostaining, cells and tissue sections were pre-incubated in 5% normal serum, and 0.025% and 0.25% Triton X-100, respectively, in KPBS for 45 min at room temperature. Incubation in primary antiserum was carried out overnight at +4°C and the following primary antibodies were used: mouse anti-β-III tubulin (1∶333, Sigma-Aldrich), goat anti-doublecortin (DCX, 1∶400, Santa Cruz Biotechnologies, Santa Cruz, USA), mouse anti-rat CD-68 (ED-1, 1∶200, AbD Serotec MorphoSys AbD, Düsseldorf, Germany), rabbit anti-GFAP (1∶500, DakoCytomation, Glostrup, Denmark), mouse anti-GFAP (1∶400, Sigma-Aldrich), mouse anti-human nucleus (HuNu, 1∶100, Chemicon, Temecula, USA), rabbit anti-Ki67 (1∶500, Abcam, Cambridge, USA), rabbit anti-human nestin (1∶500, Chemicon). Primary antibodies were detected using appropriate fluorescent Cy3 (Jackson ImmunoResearch Laboratories, West Grove, USA) or biotin-conjugated (Vector Laboratories, Burlingame, USA) secondary antibodies (1∶200), which were then detected with Alexa 488-conjugated streptavidin (1∶200, Molecular Probes). In order to determine the specificity of the primary antibodies and the level of background generated from the secondary antibodies, in one well per *in vitro*-staining the primary antibodies were omitted as negative control. For double labeling, only one biotinylated secondary antibody was used at a time. For nuclear staining Hoechst 33342 (1∶1000, Invitrogen, Carlsbad, USA) was added during final incubation with secondary antibodies. Sections were mounted on PLL-coated slides (Thermo Fisher Scientific, Waltham, USA) and slides were coverslipped with glycerol-based mounting medium. Immunostainings were controlled, microscopic images acquired and double-immunoreactivities verified with a confocal laser scanning (Leica TCS SP2, Leica Microsystems) microscope equipped with a supplementary CCD camera (Leica Microsystems).

### MRI data processing and statistical analysis

All MR images were processed with the program ImageJ (http://rsbweb.nih.gov/ij/).

For SNR analysis of the ^19^F signal, the measured signal and noise in the raw ^19^F images were corrected voxel-wise for non-Gaussian noise distribution due to the low SNR [22]. Only voxels with a corrected SNR above 3.5 were considered significant and included in the analysis.

For display purposes of the merged ^1^H/^19^F images, the fluorine SNR datasets were resized to a 128×128 matrix with bilinear interpolation, grey-levels were converted to transparency levels of red, and overlaid on the ^1^H image.

To estimate the minimum number of cells (N_min_) per voxel to generate a detectable SNR of 3.5, we assumed a linear correlation between number of cells and SNR. Knowing the summed SNR of all voxels (SNR_total_) with significant ^19^F signal and the overall number of cells (N_total_) within this volume the detection limit was then calculated with the simple relationship N_min_ = N_total_×3.5/SNR_total_, similar to a method described previously [11].

Differences between *in vitro* labeled and non-labeled cells were evaluated with Student's unpaired t-test. Data are expressed as mean±SEM and differences considered significant at p<0.05.

## Results

### Labeling of NSCs

**Figure 1:**
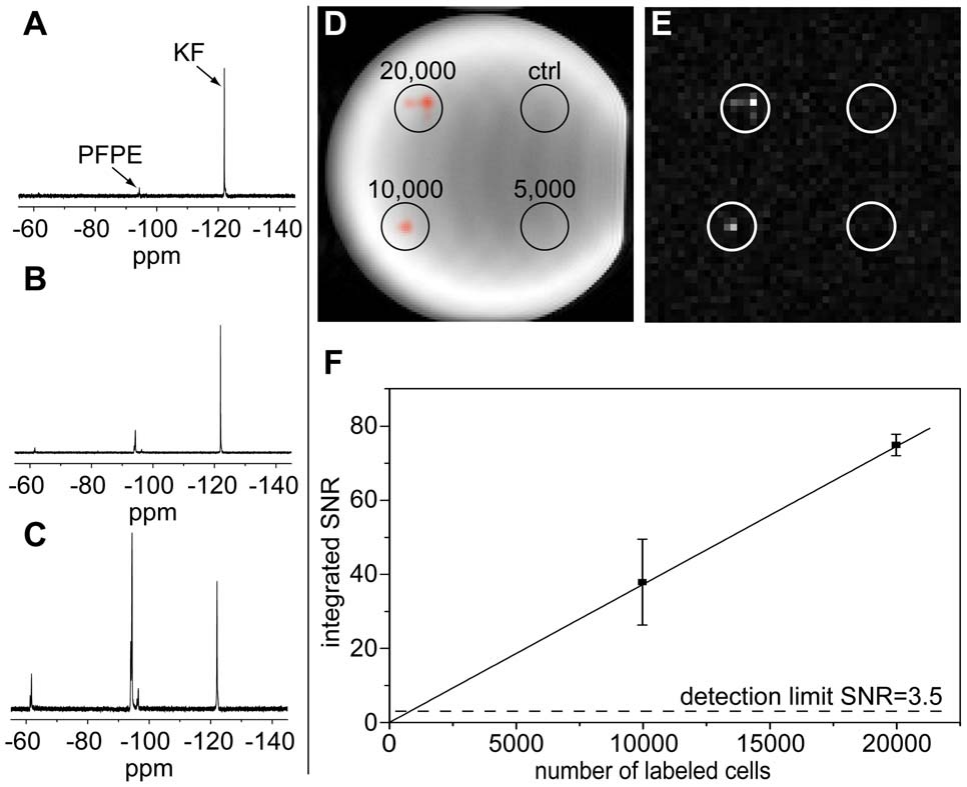
Optimization of labeling and *in vitro* detection limits. A–C: ^19^F MRS of NSCs incubated with 15 µL (A), 50 µL (B), and 100 µL (C) PFPE emulsion per mL medium together with a KF solution as an internal standard, KF frequency set to −120.9 ppm, main PFPE peak at −93.2 ppm, signal intensity in arbitrary units. The cell signal was maximized at a concentration of 100 µL/mL. D–F: MRI of 20,000, 10,000, and 5,000 labeled, and 65,000 control cells plated in gelatin, D shows the merged ^1^H and ^19^F, E the ^19^F image only. The 20,000 and 10,000 cells spots were clearly detectable, whereas a significant signal was not detected for the 5,000 cells, probably due to cells spreading over many voxels, thus not exceeding the critical detection limit. From the summed SNR of the two spots with ^19^F signal we estimate that approximately 1,000 cells need to accumulate in one image voxel to overcome the detection limit, as indicated by the crossing of the linear fit in F with the dashed line illustrating an SNR of 3.5. For the fit we assumed 0 SNR for 0 cells. Error bars in F are over n = 3 technical replicates. Note: The surface coil was adjusted parallel to the paper plane.

NSCs readily took up the PFPE emulsion without the aid of transfection agents. The intracellular ^19^F content was maximized for 100 µL of the ^19^F marker substance per mL medium as confirmed with MRS (Fig. 1, A–C). With this protocol labeled cells contained on average 3.70±0.78*10^12^ (n = 3 biological replicates) ^19^F spins.

In order to analyze if the cells keep the fluorine marker over time, we took out 1/3 of the cells (i.e. 1/3 of the internalized ^19^F) directly after the labeling procedure and determined the amount of intracellular ^19^F with MRS. The remaining 2/3 of the cells were replated for another 7 days. The incorporated ^19^F was then again measured and 2.10±0.54 times (n = 4 technical replicates) the amount of the first sample was found, which is in good agreement with the expected value of 2 in case there was no significant loss of label.

The measured relaxation times were T_1_/T_2_ = (280±20) ms/(153±4) ms for the free PFPE marker and T_1_/T_2_ = (380±4) ms/(68±3) ms for the labeled cells.

### Detectability and quantification in vitro

MRI of a dilution series of labeled NSCs (20,000, 10,000, 5,000 labeled and 65,000 non-labeled cells for control) suspended in gelatin clearly detected spots with 20,000 down to 10,000 cells, while there was no background ^19^F signal from non-labeled cells. Spots with 5,000 cells could not be detected by *in vitro* MRI. From the SNR of the two cell clusters with significant signal (SNR_total_ = 112±14, n = 3 technical replicates) we estimate that the minimum number of cells, which need to accumulate in one image voxel in order to generate significant signal *in vitro*, is approximately N_min_≈1,000 cells (Fig. 1, D–F). This finding was supported by ^19^F MRI of a dilution series of the ^19^F marker (supplementary Fig. S1). Although the 5,000 cell spot is clearly above the estimated detectable number of cells, it was most likely not discovered because cells were spread over too many image voxels.

### Effect of the ^19^F marker in vitro

**Figure 2:**
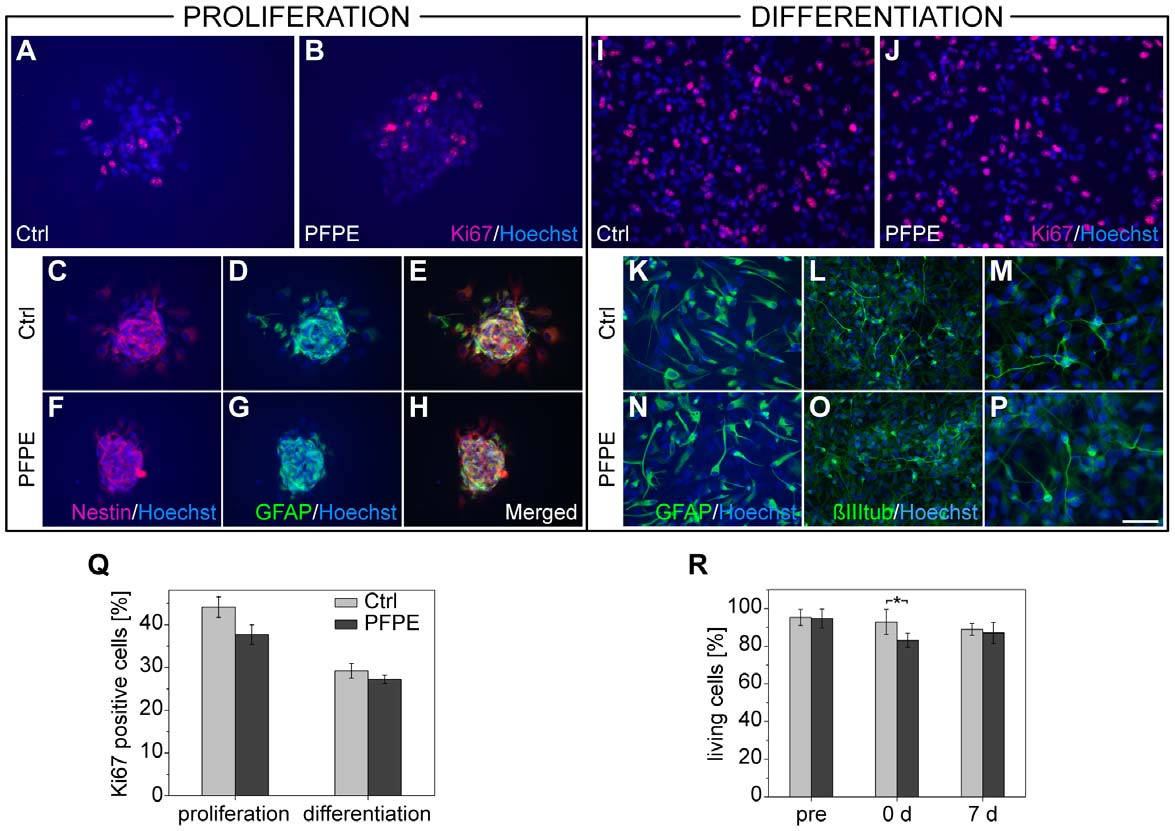
Effect of the labeling on cell viability, proliferation, and differentiation capacity. Photomicrographs of *in vitro* samples stained for Ki67 (A, B, I and J), nestin (C, F E and H), GFAP (D, E G, H, K and N), and βIII-tubulin (L, M, O and P). There were no major qualitative differences in the viability or proliferation and differentiation capacity of the labeled (B, F, G, H, J, N, O and P) versus non-labeled control cells (A, C, D, E, I, K, L and M), both during proliferation (A–H), i.e. directly after the labeling procedure, and after 9 days of differentiation (I–P). The percentage of proliferating Ki67+ cells during proliferation or after differentiation was not significantly altered by the labeling (Q). Quantification of the number of living cells before, 0 days after, and 7 days after incubation with the ^19^F marker revealed that the viability was significantly decreased compared to controls at 0 days but normalized at 7 days after labeling (R). The scale bar represents 25 µm for M and P, 50 µm for all others.

The labeling procedure had only very minor effects on the cells' viability and no significant effects on their proliferation (Fig. 2, A–H) and differentiation (Fig. 2, I–P) capacity. Furthermore, carefully analyzing both the proportion and morphology of nestin+, GFAP+ and β-III tubulin+ cells, we did not detect any significant changes compared to the non-labeled control cells. Also, no differences in the numbers of Ki67+ cells were found either during proliferation (37.7±3.2%; directly after the labeling) or after 7 days of differentiation (27.2±1.0%) *in vitro* compared to non-labeled control cells (44.1±2.4% for non-differentiated and 29.2±1.7% for differentiated cells, n = 3 technical replicates) (Fig. 2, Q).

The percentage of living cells was equal compared to controls before labeling (95.2±4.2% and 94.7±5.0%) but significantly decreased directly after incubation with the ^19^F marker (83.1±3.7% compared to 92.9±6.6% for unlabeled cells, p<0.05). However, this value normalized again after replating and subsequent culturing of the cells for 7 days (87.0±5.6% for the labeled cells and 88.9±3.2% for controls, n = 3 biological replicates, Fig. 2, R).

### ^19^F signal detectability in vivo

**Figure 3:**
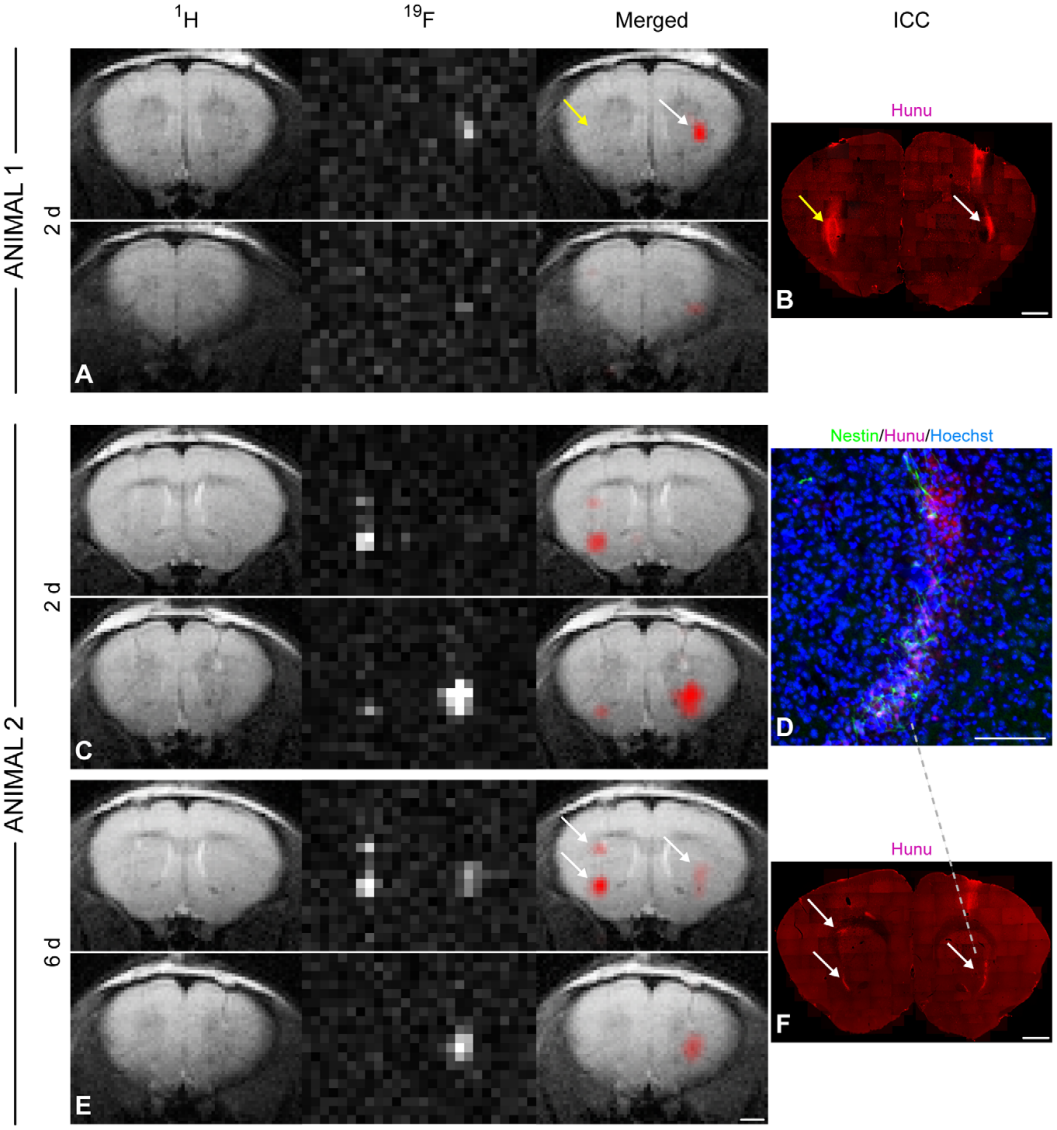
*In vivo* ^19^F MRI and correlation with immunohistochemistry. ^1^H, ^19^F, and merged MR images of a mouse (Animal 1), which had been injected with non-labeled control cells into the left striatum and labeled NSCs into the right hemisphere (A). Only the labeled cells generated a ^19^F signal whereas hunu staining confirmed the presence of cell grafts on both sides as indicated by the arrows (B). MRI of another mouse (Animal 2) 2 days (C) and 6 days (E) after grafting showed no major signal loss in the ^19^F images over time. This animal had received two deposits of labeled cells in the left striatum and one deposit in the right striatum. The location and intensity of ^19^F signal from cell clusters, marked with white arrows, correlated well with hunu staining on histological sections. Note that the ^19^F resolution allows the distinction of the two clusters on the left hemisphere (B, F). Only cells that were clearly immunoreactive to both hoechst and hunu were considered as grafted human NSCs (D). Scale bars are 50 µm for D, 1 mm for all others.

In order to assess whether ^19^F MRI is a useful tool for the longitudinal tracking of implanted cells in the brain, we injected PFPE labeled NSCs and non-labeled control cells into the striatum of mice and scanned the animals a few days later. We detected significant ^19^F signal from the grafts with labeled cells in all animals, while there was no signal from implanted non-labeled control cells (Fig. 3, A). Presence of Hoechst+/hunu+ cells allowed definite assignment of NSC aggregations on histological sections (Fig. 3, B, D, and F). These cell spots corresponded well, both in intensity and location, to regions exhibiting significant ^19^F signal. In the two mice with labeled cells grafted on both hemispheres, the ^19^F signal persisted at least 6 days after implantation (Fig. 3, C and E). Quantitative SNR analysis of these animals revealed that total ^19^F SNR decreased by ≈20% from day 2 to day 6.

To estimate the *in vivo* detection limit N_min_ at 2 days after surgery, the overall ^19^F SNR in all animals was determined (SNR_total_ = 612.5) and used together with the known number of transplanted cells (N_total_ = 1.5 million) to calculate the minimum number of detectable cells at an SNR = 3.5 (N_min_≈9,000).

### Effect of the ^19^F marker in vivo

**Figure 4:**
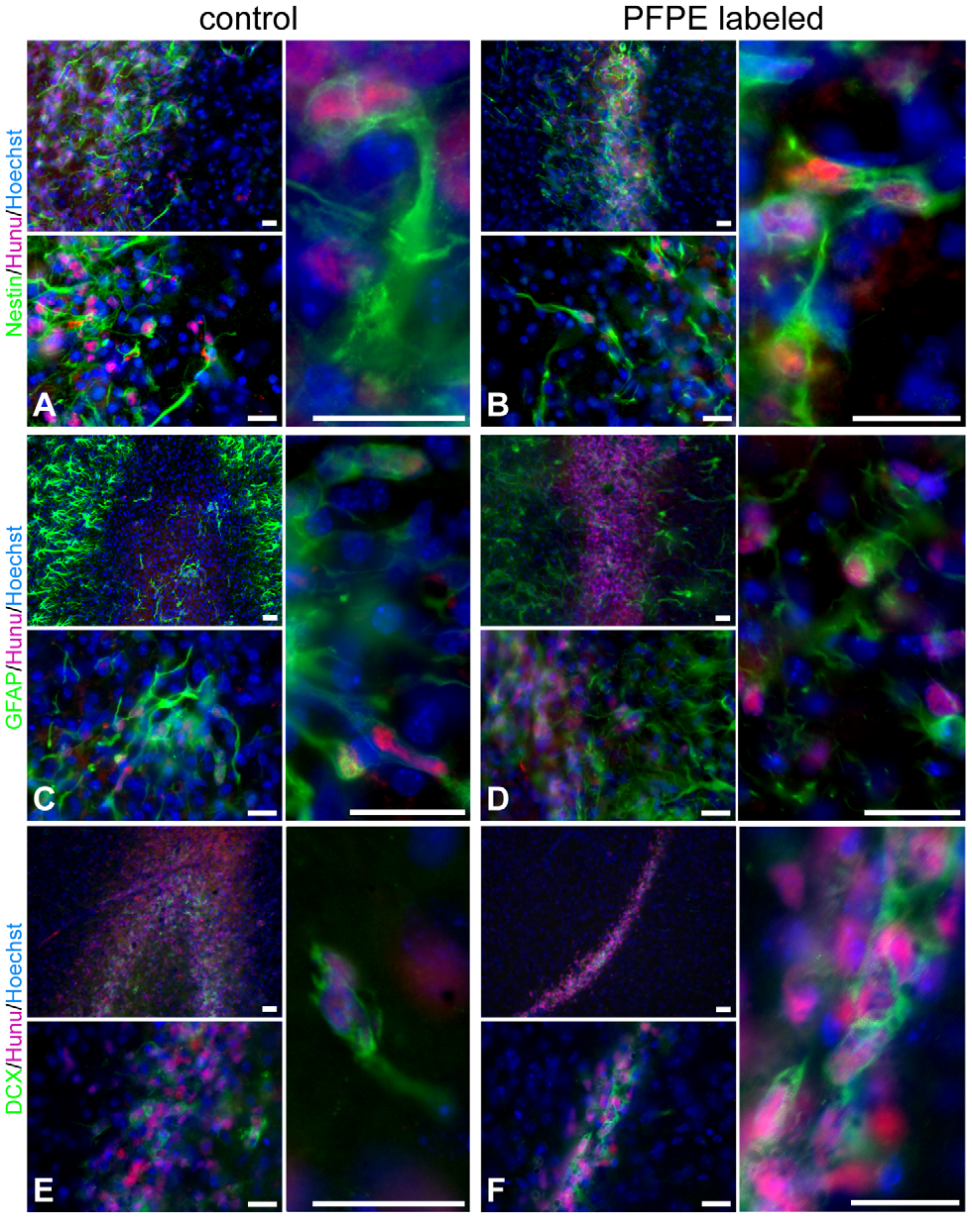
Effect of the ^19^F marker on cell phenotypes *in vivo*. Photomicrographs of non-labeled control (A, C, E) and PFPE labeled NSCs (B, D, F) on tissue sections from transplanted animals. The majority of the implanted cells were, at this early time point after injection, still neural stem and progenitor cells as confirmed by nestin/hunu and GFAP/hunu stainings (A–D). Presence of DCX+/hunu+ (E, F) neuroblasts showed that NSCs were capable of neuronal differentiation. By qualitative analysis we did not detect major differences in the PFPE labeled cell population compared to control cells. Scale bars are 20 µm.

At the end of the ^19^F MRI *in vivo* measurements, the animals were sacrificed and the brains were processed for immunohistochemistry to study implanted cell phenotypes. Qualitative analysis of brain sections stained for nestin, GFAP and DCX showed that the vast majority of the implanted NSCs were nestin+ and GFAP+ cells (Fig. 4 A–D). We also detected a portion of the implanted cells that had already differentiated into DCX+ neuroblasts (Fig. 4 E and F). Overall, these images revealed that the fraction of cells of each cell phenotype studied was similar for ^19^F labeled and non-labeled cell grafts.

## Discussion

We show that efficient labeling of NSCs with a PFC-based marker is possible without impairment of their naïve cell behavior and thus, in consequence, their therapeutic potential. We have successfully demonstrated detectability of these human neural stem cells with high sensitivity after implantation into mouse brain and the usefulness of this imaging approach for monitoring the cell dynamics longitudinally because the graft was followed with ^19^F MRI for a week, being most likely detectable even much longer.

### Efficacy and effects of labeling NSCs with ^19^F

PFC nano-emulsions are particularly suitable agents for stem cell tracking because of the excellent MR properties for imaging and the high biocompatibility [10], [23]. Important for a possible clinical translation, PFCs are chemically stable, biologically inert, and related compounds have already been evaluated in preclinical and clinical trials as blood-substitutes [23], [24], [25].

Our labeling procedure with PFPE lead to an uptake of 3.7*10^12^ spins/cell, similar to reports on other cell types. For experimental studies, ^19^F uptake could potentially be further increased through the use of transfection agents, however, most of these are not approved for use in the clinic [10]. We found a high T_2_/T_1_ ratio both for the free PFPE tracer and for the labeled cells. This indicates that the MR properties of the tracer are in deed optimal in terms of maximizing the SNR particularly when using turbo spin echo sequences. We note however, that the reported relaxation times may not be universal, e.g. due to slice thinning or excitation/refocusing flip angle imperfections and echo train signal variations.

Careful assessment of important immunohistochemical markers revealed that the label has only minor effects on viability, differentiation capacity, and proliferation *in vitro* and *in vivo*. In other reports the viability of PFC labeled stem cells was not different from non-labeled controls [11], [20] whereas we observed a decrease in viability directly after incubation. This could either be a specific feature of the human NSCs or be due to the relatively high concentration of ^19^F marker in the medium compared to those other studies. We observed a decrease in viability over time, although non-significant, also in the non-labeled control cells. This suggests that the impairment in viability may rather be related to other factors such as the PolyHEMA coating of flasks.

The use of a fluorescent agent, attached to the PFPE, could provide a more direct proof that the agent is internalized, analyzing localization by fluorescence microscopy. However, most fluorophores are not suitable for clinical use and, therefore, we used an emulsion without fluorescent tag. We included thorough washing steps in all procedures in order to remove excessive ^19^F marker particles. Moreover, a study with a similar, fluorescent PFC emulsion showed localization of the ^19^F marker in the cytosol of murine neural progenitor cells and persistence of ^19^F MRI signal over 2 weeks [20]. Importantly, we did not detect a decrease in MRS-determined ^19^F content from labeled, replated cells over a period of one week. *In vivo* the ^19^F SNR only slightly decreased between day 2 and day 6 after implantation. Overall, there is strong evidence that NSCs retain the label over time and can be tracked longitudinally, even for longer periods than the time span of this study. This feature of long-term tracking of cells was not provided employing alternative fluorine markers such as Poly-L-lysine CF_3_, for which labeled cells lost half the ^19^F signal within 7 days [26], [27]. However, thorough long-term evaluations of both effects of PFCs on grafted stem cells and efficacy of the labeled cells in the pathological brain e.g. stroke models are needed in the future.

### Cell quantification and detection limits

The ^19^F MR signal is proportional to the amount of magnetically equivalent fluorine nuclei within a certain voxel. This allows quantification of the number of cells. If the total number of implanted cells is known, one strategy is to link this number to the sum total of SNR and to assume linear correlation between both. This way each SNR can be assigned a cell number. We employed this for the determination of detection limits by extrapolation of the number of cells generating an SNR of 3.5. However, the strategy works only under the assumption that cells are labeled homogeneously, do not lose the label, and do not proliferate as this leads to a dilution of ^19^F content/cell, and that partial volume effects can be neglected. The assumption of a homogeneous labeling is reasonable when fluorine content per cell is averaged over a few hundred cells. Further, we have shown containment of the ^19^F marker within NSCs over one week under *in vitro* conditions. The dilution of label through cell division is a potential threat to our strategy, since injected cells can divide after transplantation. However, the cells used here cease to proliferate shortly after implantation. The previously reported time span of 2 days for proliferation *in vivo* is rather short considering a doubling time of approximately 72 hours under ideal *in vitro* conditions [18], [19], [28], [29].

With the small graft sizes used in brain implantation studies, an image voxel is typically only partly filled by labeled cells. This becomes particularly severe with ^19^F MRI as one strategy to enhance sensitivity is to measure with rather low spatial resolution [10]. As a consequence, quantification in regions with cell densities below the detection limit leads to underestimated cell numbers, graft borders are only inaccurately defined on ^19^F MR images, and migratory processes on a small scale cannot be monitored. A post-processing scheme to ease the problem has been applied in models of T-cell homing to the pancreas or lymph node by dilation of regions of interest by one-half voxel [12], [13]. This scheme could potentially be included in future MRI studies of ^19^F labeled NSCs depending on expected cell distribution. In order to minimize these partial volume effects in the first place, we used a relatively high *in vivo* resolution (160 nL voxel size in the present report compared to for example 305 nL used before in the mouse brain [20]). To our knowledge there is only one *in vivo* study with a higher resolution, but in mouse hind limb (15.6 nL voxel size [30]). To compensate the loss of sensitivity at the higher resolution, we decided for the use of surface radiofrequency coils with inhomogeneous field profile and T_2_-weighted turbo spin echo pulse sequences. Thus the measured ^19^F signal from a certain region depended not only on the amount of fluorine contained therein but also on ^19^F relaxation times and the region's distance from the coil. The use of time-consuming, purely spin-density weighted pulse sequences can improve quantification only at the cost of SNR per unit time. A volume coil with more uniform magnetic field profile may be better suited for quantification. However, the coil is often larger and less sensitive compared to a surface coil. To minimize the problem we adjusted the setup in a way that the cell spots were placed within the same distance from the coil for the *in vitro* experiments. For the *in vivo* experiments cells were spread over a slightly larger region and further away from the coil, thereby leading to loss of signal. This may partly explain the order of magnitude difference of *in vitro* and *in vivo* detection limits. In conclusion, coil design and choice of pulse sequence must be carefully adapted to the specific application in future studies employing ^19^F MRI for cell tracking.

Using a ^19^F marker with high spin density, using optimized radiofrequency coils and a very high field of 11.7 T we show detection of small numbers of cells in a preclinical setting. Our data indicates that within ∼1 h acquisition time *in vitro* detection of ∼1,000 cells/voxel is feasible corresponding to less than 10^16^ fluorine spins/voxel. *In vivo* we estimate a detection limit in the order of ∼10,000 cells/voxel. The limits are in agreement with earlier studies [11], [12], [14]. These numbers are usually sufficient for imaging the graft core and possibly even for larger cell clusters migrating away from injection sites in the brain.

### Potential of ^19^F MRI of NSCs in cell replacement therapy

The usefulness of cell tracking with ^19^F MRI mainly depends on its sensitivity. Although the ^19^F marker and MRI methods used in this study are likely to be safe for clinical translation, detection sensitivity will be orders of magnitude lower in a clinical setting due to lower field strengths, larger radiofrequency coils, and shorter acquisition times [31]. Still, ^19^F MRI of NSCs may become clinically relevant in the near future as a result of further advances in MRI hardware and pulse sequence development.

However, before stem cell based therapy of neurological disorders can be used in human patients the action of these cells needs to be further investigated in animal models [16]. ^19^F labeling may prove superior to labeling with magnetic iron oxide nanoparticles in such preclinical investigations particularly when the pathology induces changes in ^1^H relaxation times that make distinction between diseased tissue and labeled cells difficult. Possible scenarios include vasogenic edema after ischemic stroke or lesions involving bleedings, in which heterogeneous background T_1_, T_2_, and T_2_* impose major challenges in unambiguous detection of magnetically labeled cells with ^1^H MRI. As ^19^F MRI allows quantification of cell numbers it may also help to optimize efficacy of stem cell therapy e.g. in terms of injection sites, monitoring graft size changes [29] and routes of migration.

### Conclusion

In future clinical applications, NSCs are one of the most potential and attractive cell sources for stem cell based treatment of diseases affecting the central nervous system. However, for the successful translation of cell therapy additional live imaging tools such as MRI are a prerequisite. ^19^F MRI is a promising, quantitative, and non-invasive technique to monitor NSC grafts after implantation. We have shown that labeling NSCs with a PFC marker, potentially suitable for clinical use, does not significantly impair cell function both *in vitro* and *in vivo*. Our high detection sensitivity and resolution have allowed for the first time true *in vivo* tracking of small groups of human NSCs. This is particularly interesting for studies of brain cell replacement therapy, in which the graft can initially be small and may become further diluted through lesion-induced cell migration.

## Supporting Information

Figure S1 **Dilution series of ^19^F marker.** ^19^F MRI of six tubes containing different concentrations of the PFPE agent (1, 2, 3, 4, 5, and 6 times 31.1 mM corresponding to multiples of 3*10^15^ ^19^F spins/voxel). The tube with 3*10^15^ spins/voxel can clearly be depicted (SNR∼6). Assuming a labeling efficacy of 3–4*10^12^ spins/cell this would translate to detection of less than 1,000 cells/voxel in agreement with the results obtained with our quantification strategy (Fig. 1). Pulse sequence parameters were chosen identical to the cell dilution series in Fig. 1. Note: The surface coil was oriented parallel to the paper plane.
(TIF)
