# Supplementary figures and images for: In Vivo Tracking of Human Neural Stem Cells with 19F Magnetic Resonance Imaging
Source: PLoS One. 2011 Dec 28;6(12):e29040. doi: 10.1371/journal.pone.0029040 (PMC3247235; doi:10.1371/journal.pone.0029040)

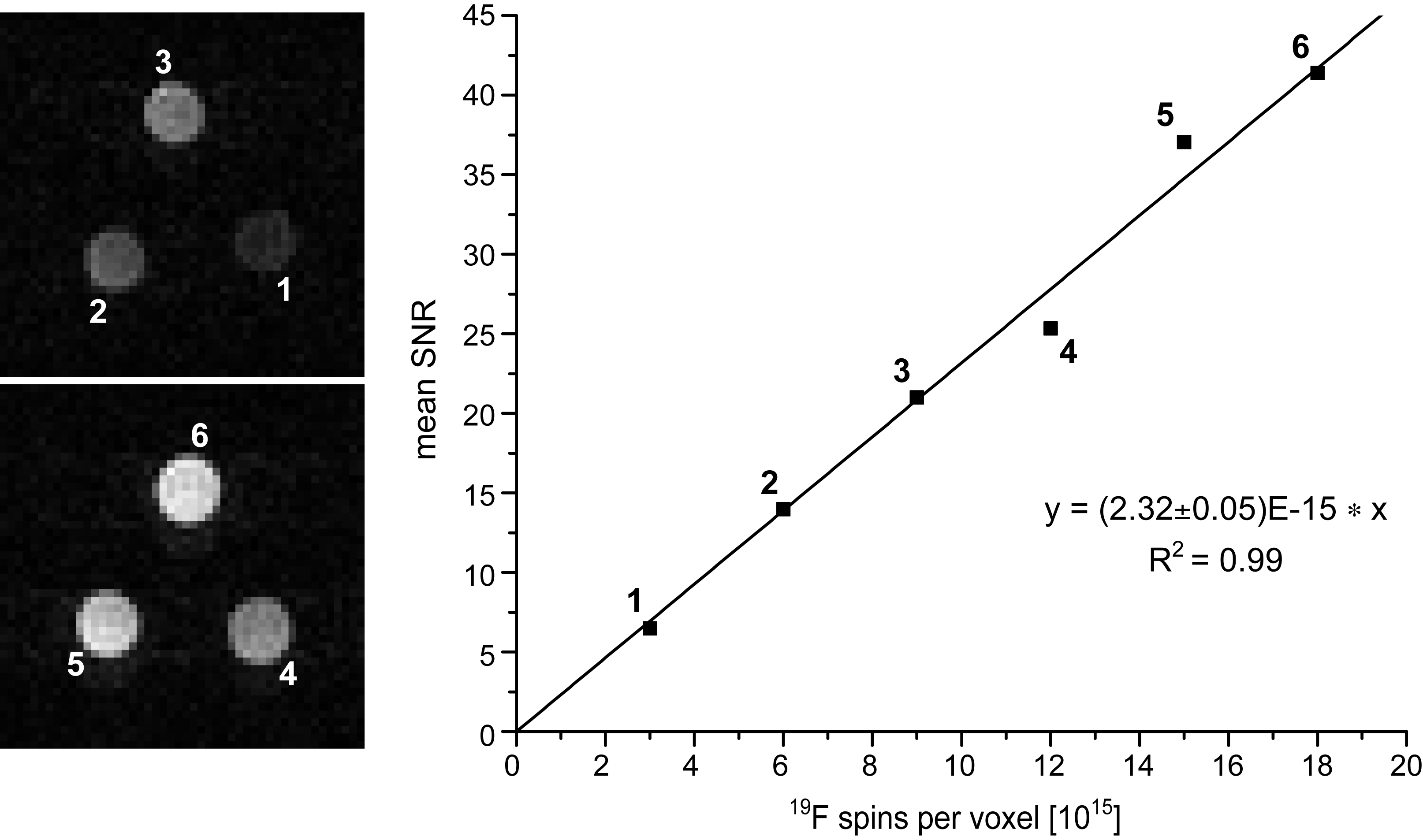

Supplement: Figure S1 — Dilution series of 19F marker. 19F MRI of six tubes containing different concentrations of the PFPE agent (1, 2, 3, 4, 5, and 6 times 31.1 mM corresponding to multiples of 3*1015 19F spins/voxel). The tube with 3*1015 spins/voxel can clearly be depicted (SNR∼6). Assuming a labeling efficacy of 3–4*1012 spins/cell this would translate to detection of less than 1,000 cells/voxel in agreement with the results obtained with our quantification strategy (Fig. 1). Pulse sequence parameters were chosen identical to the cell dilution series in Fig. 1. Note: The surface coil was oriented parallel to the paper plane. (TIF) [file pone.0029040.s001.tif]
